# Documented penicillin allergy and beta-lactam antibiotic use in Massachusetts long-term care facilities: opportunities for penicillin allergy delabeling

**DOI:** 10.1017/ash.2024.434

**Published:** 2024-10-08

**Authors:** Kap Sum Foong, Shira Doron, Leslie Fowle, Melissa Cumming, Jessica Leaf, Barbara Bolstorff, Christina Brandeburg, Ye Chen, Alysse Wurcel

**Affiliations:** 1 Division of Geographic Medicine and Infectious Diseases, Tufts Medical Center, Boston, MA, USA; 2 Division of Epidemiology, Bureau of Infectious Disease and Laboratory Sciences, Massachusetts Department of Public Health, Boston, MA, USA; 3 Tufts Clinical and Translational Science Institute, Tufts School of Graduate Biomedical Sciences, Boston, MA, USA

## Abstract

In a cross-sectional study of 20 Massachusetts long-term care (LTC) facilities, 19% (n = 449) of residents received antibiotics, with approximately one-third having a documented penicillin allergy. This documented allergy decreased the likelihood of prescribing beta-lactam antibiotics for infections. Improved penicillin allergy assessments and delabeling could optimize antibiotic prescribing in LTC settings.

## Introduction

Long-term care (LTC) residents experience high mortality rates from infections.^1^ Approximately 50% to 75% of LTC residents in the United States (US) receive at least one antibiotic annually, predominantly fluoroquinolones.^2^ Optimizing antibiotic use is crucial for managing these infections effectively, but this effort is often compromised by inaccurate penicillin allergy labels. Such labels frequently lead to the selection of broader-spectrum antibiotics, which can contribute to antimicrobial resistance, increased risk of side effects, and higher healthcare costs.^3^

A recent study found nearly one in four residents in twenty LTC facilities had a documented penicillin allergy, a rate significantly higher than the one in ten observed in the general US population.^4^ Inaccurate penicillin allergy labeling poses a critical health threat in this vulnerable population because older individuals are more susceptible to mortality from multidrug-resistant infections and adverse effects from broad-spectrum antibiotics.^5,6^

Although it is known that patients with a penicillin allergy are less likely to receive beta-lactam antibiotics, existing studies have often overlooked the LTC population. The high rates of fluoroquinolone prescriptions in LTC settings highlight a significant knowledge gap.^2^ Understanding the factors driving these prescribing trends and the context-specific differences across clinical settings is crucial. The goal of this study is to measure the impact of electronic health record (EHR)-documented penicillin allergies on the prescribing patterns of beta-lactam antibiotics in Massachusetts LTC populations. This understanding can inform penicillin allergy delabeling efforts, which are currently lacking in LTC settings.

## Methods

In partnership with the Massachusetts Department of Public Health (MDPH), we conducted a cross-sectional study involving 20 LTC facilities. Each LTC facility provided a de-identified census of all residents present on the designated point prevalence survey day, which took place only once on a single day between July 1, 2023, and March 31, 2024. These data included demographics, penicillin allergy records, antibiotic prescriptions on the survey date and within the preceding 30 days, and indications for antibiotic use based on EHR documentation. The research team (KSF) entered these de-identified data into a central database using the Research Electronic Data Capture tool, as previously described.^4^

We divided the antibiotics into two principal groups: beta-lactams (including penicillins, cephalosporins, and carbapenems) and non-beta-lactams. Indications for antibiotics were categorized into infection sites, including the urinary tract, respiratory tract, and skin and soft tissue. All other or unspecified indications were grouped into an “others” category.

The primary exposure was an EHR-documented allergy to any penicillin antibiotic. The primary outcome was the prescription of beta-lactam antibiotics, categorized by indications (all indications, urinary tract infections, respiratory tract infections, and skin and soft tissue infections [SSTI]).

We used multivariable regression analysis to explore the association between documented penicillin allergy and the prescription of beta-lactam antibiotics, categorized by indications (ie, urinary tract infections, respiratory tract infections, and SSTI). The model adjusted for covariates such as age, sex, race, and allergies to non-penicillin antibiotics. We report adjusted odds ratios (aORs) with 95% confidence intervals (CIs). Statistical analyses were conducted using SPSS, version 25 (IBM, Armonk, NY, USA). All *p* values were from 2-sided tests, with results deemed statistically significant at *p* ≤.05. This study was approved by the Institutional Review Boards of Tufts Medical Center and the MDPH, which determined it was not human subjects research and individual informed consent was therefore not required.

## Results

Of the 2,345 LTC residents, 449 (19.1%) entered the analytic cohort because they received antibiotics (Supplemental Table 1). Among these residents, 34.7% (n = 156) had a documented penicillin allergy. Most residents (86.2%, n = 387) received a single antibiotic, while 13.6% (n = 61) received two antibiotics. The primary indications for antibiotic prescriptions were urinary tract infections (45.4%, n = 204), respiratory tract infections (29.2%, n = 131), and SSTI (18.5%, n = 83). Beta-lactams accounted for 45.5% of the 512 antibiotic prescriptions. Further details on the distribution of prescribed antibiotics are provided in Table 1.

**Table 1.** Antibiotic prescriptions among long-term care residents by documented penicillin allergy status

Antibiotic	All antibiotic prescriptions (*n* = 512), *n* (%)	Antibiotic prescriptions for residents with a documented penicillin allergy (*n* = 171), *n* (%)	Antibiotic prescriptions for residents without documented penicillin allergy (*n* = 341), *n* (%)
Beta-lactams (*n* = 233)			
Penicillins	66 (12.9)	0	66 (19.3)
First generation cephalosporins	56 (10.9)	9 (5.3)	47 (13.7)
Second generation cephalosporins	3 (0.6)	0	3 (0.9)
Third generation cephalosporins	101 (19.6)	12 (6.5)	89 (26.3)
Fourth generation cephalosporins	2 (0.4)	0	2 (0.6)
Carbapenems	5 (1.0)	0	5 (1.4)
Non-beta-lactams (*n* = 279)			
Fluoroquinolones	94 (18.4)	66 (38.8)	28 (8.2)
Doxycycline	56 (10.9)	29 (17.1)	27 (7.9)
Macrolides	34 (6.6)	17 (10.0)	17 (5.0)
Nitrofurantoin	31 (6.1)	13 (7.6)	18 (5.3)
TMP-SMX	29 (5.7)	17 (10.0)	12 (3.5)
Clindamycin	7 (1.4)	2 (1.2)	5 (1.5)
Others^a^	28 (5.5)	6 (3.5)	22 (6.4)

TMP-SMX, trimethoprim-sulfamethoxazole

a Others included daptomycin (*n* = 2), fosfomycin (*n* = 2), fidaxomicin (*n* = 1), linezolid (*n* = 4), metronidazole (*n* = 5), intravenous vancomycin (*n* = 5), oral vancomycin (*n* = 9).

**Figure 1.**
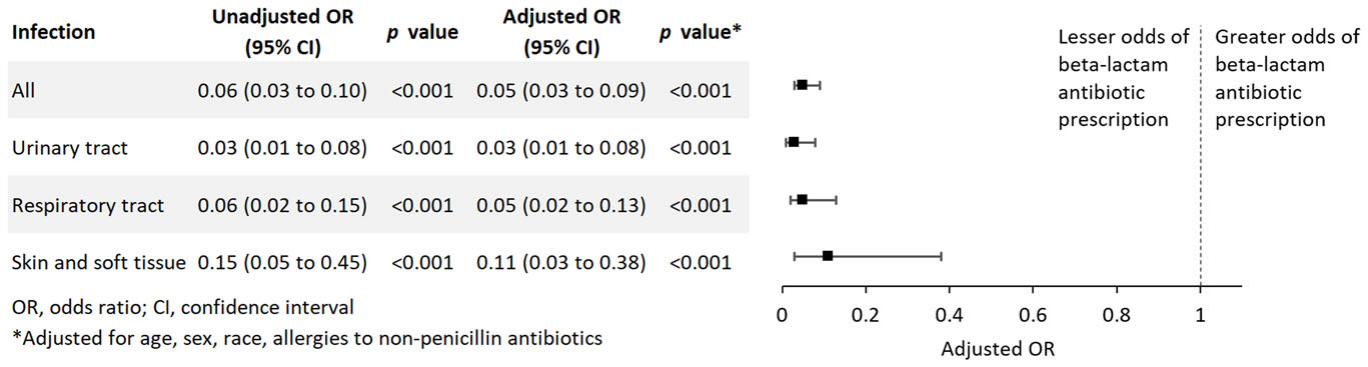
Multivariable assessment of the association of documented penicillin allergies with beta-lactam antibiotic use for all infections and specified infections among long-term care residents.

Residents with a documented penicillin allergy were significantly less likely to be receiving beta-lactam antibiotics for all infections (adjusted odds ratio [aOR], 0.05; 95% confidence interval [CI], 0.03 to 0.09), urinary tract infections (aOR, 0.03; 95% CI, 0.01 to 0.08), respiratory tract infections (aOR, 0.05; 95% CI, 0.02 to 0.13), and SSTI (aOR, 0.11; 95% CI 0.03 to 0.38) (Figure 1).

## Discussion

In Massachusetts LTC facilities, penicillin allergies are prevalent among residents receiving antibiotics. Residents with documented penicillin allergies were 95% less likely to receive beta-lactam antibiotics for any infection. Beta-lactam antibiotics are the preferred treatment for many infectious syndromes, including respiratory tract infections and SSTI. However, their use is notably lower in many LTC patients. The use of alternative antibiotics often leads to suboptimal treatment outcomes, increased antibiotic use, and a higher risk of antimicrobial resistance.^2,3^

The US Centers for Disease Control and Prevention recommends penicillin allergy evaluation as part of hospital antimicrobial stewardship programs to optimize antibiotic use. In addition, the American Academy of Allergy, Asthma, and Immunology’s Drug Allergy Practice Parameter Update of 2022 advocates for proactive penicillin allergy delabeling across all populations. Despite these endorsements, a survey of 121 US hospitals revealed limited access to penicillin allergy assessment in inpatient settings.^7^ Our previous study identified significant deficiencies in the documentation of antibiotic allergies in LTC residents.^4^ Currently, there is no standardized method for implementing penicillin allergy assessment in LTC settings. Barriers such as resource constraints and high staff turnover further complicate the implementation of effective penicillin allergy delabeling strategies in LTC facilities.

Simplified, evidence-based pathways and tools have been developed for use by non-allergists in acute care settings.^8,9^ These tools consider the initial allergic reaction history and the time elapsed to identify low-risk individuals for delabeling.^8,9^ Remarkably, about 20% of documented penicillin allergies can be delabeled based solely on allergy history, including cases with a family history of penicillin allergy without a personal history of allergic reactions, and those with intolerance reactions inconsistent with immune-mediated responses.^10^ Given their simplicity and low resource requirements, these pathways are particularly viable for implementation in resource-limited settings such as LTC facilities compared to more resource-intensive methods like oral challenges or penicillin skin testing. However, assessment tools designed for penicillin allergy delabeling, such as the PEN-FAST tool, do not adequately account for conditions like dementia or cognitive impairment, complicating the assessment and management of penicillin allergies in LTC populations.^9^

This study has several limitations, including its cross-sectional design, which prevents causality inference. The geographical scope, limited to Massachusetts and the inclusion of 20 LTC facilities, restricts the generalizability of the findings. Furthermore, we could not determine the duration of antibiotic use, nor could we use clinical and culture data to assess the appropriateness of antibiotic use. Adjustments for demographic and medical variables may not fully account for all potential confounding factors, such as local antimicrobial resistance patterns and institutional prescribing policies, which can influence antibiotic selection in LTC facilities.

In conclusion, our data demonstrate that LTC residents with documented penicillin allergies are significantly less likely to receive beta-lactams for common infections. This underscores the urgent need to improve penicillin allergy assessments and delabeling strategies in LTC settings.

## Supporting information

Foong et al. supplementary material Foong et al. supplementary material

## Supplementary material

For supplementary material accompanying this paper visit http://doi.org/10.1017/ash.2024.434. click here to view supplementary material
