# Supplementary material for: Documented penicillin allergy and beta-lactam antibiotic use in Massachusetts long-term care facilities: opportunities for penicillin allergy delabeling
Source: Antimicrob Steward Healthc Epidemiol. 2024 Oct 8;4(1):e166. doi: 10.1017/ash.2024.434 (PMC11488468; doi:10.1017/ash.2024.434)
Supplement: Foong et al. supplementary material [file S2732494X24004340sup001.docx]

**Supplemental Table 1.** Comparison of demographic characteristics, number of antibiotic prescriptions, and antibiotic indications among 449 long-term care residents, by documented penicillin allergy status

| Characteristics | All residents who received antibiotics  (n=449), n (%) | Residents with a documented penicillin allergy (n=156), n (%) | Residents without documented penicillin allergy (n=293), n (%) |
| --- | --- | --- | --- |
| Age in years, mean (SD) | 81.5 (12.1) | 82.4 (11.6) | 81.0 (12.3) |
| Sex |  |  |  |
| Female | 312 (69.5) | 114 (73.1) | 198 (67.6) |
| Male | 137 (30.5) | 42 (26.9) | 95 (32.4) |
| Race |  |  |  |
| White | 422 (94.0) | 150 (96.2) | 272 (92.8) |
| Non-white | 27 (6.0) | 6 (3.8) | 21 (7.2) |
| No. of antibiotic prescriptions |  |  |  |
| 1 | 387 (86.2) | 141 (25.8) | 246 (13.7) |
| 2 | 61 (13.6) | 15 (2.8) | 46 (2.6) |
| 3 | 1 (0.2) | 0 | 1 (0.06) |
| Indications^a^ |  |  |  |
| Urinary tract infections | 204 (45.4) | 78 (14.4) | 126 (7.0) |
| Respiratory tract infections | 131 (29.2) | 53 (9.8) | 78 (4.3) |
| SSTI | 83 (18.5) | 22 (4.1) | 61 (3.4) |
| Others^b^ | 39 (8.7) | 7 (1.3) | 32 (1.8) |

No., number; SD, standard deviation; SSTI, skin and soft tissue infections

^a^Eight residents had two different indications

^b^Others included *Clostridioides difficile* (n=9), intraabdominal infection (n=7), bloodstream infection (n=6), bone and joint infection (n=5), all others or unspecified (n=12)
